# Short-term effect of oral glucose solution in older patients with hypoglycemia undergoing hemodialysis

**DOI:** 10.3389/fendo.2026.1861404

**Published:** 2026-06-19

**Authors:** Jinzhu Li, Xin Shen, Jie Zhang, Xiaoling Cheng, Xian Zhang, Yuanda Wang, Guang Yang

**Affiliations:** 1 Department of the Sixth Health Care, The Second Medical Centre & National Clinical Research Centre, Chinese People’s Liberation Army (PLA) General Hospital, Beijing, China; 2 Department of Geriatric Nephrology, The Second Medical Centre & National Clinical Research Centre, Chinese People’s Liberation Army (PLA) General Hospital, Beijing, China; 3 Department of Endocrinology, The Second Medical Center & National Clinical Research Center, Chinese People’s Liberation Army (PLA) General Hospital, Beijing, China; 4 Department of the Sixth Health Care, The Sixth Medical Centre, Chinese People’s Liberation Army (PLA) General Hospital, Beijing, China; 5 Department of Nephrology, The First Medical Centre, Chinese People’s Liberation Army (PLA) General Hospital, Beijing, China

**Keywords:** continuous glucose monitoring, hemodialysis, hypoglycemia, hypotension, older

## Abstract

**Background:**

Hypoglycemia is a common complication that is closely associated with poor prognosis in older patients with diabetes undergoing hemodialysis (HD). However, evidence regarding simple intradialytic preventive strategies remains limited. We evaluated the short-term effects of 15 g oral glucose solution on blood glucose levels and blood pressure.

**Methods:**

This single-arm, self-controlled, pre-post pilot study included 12 older patients with diabetes undergoing HD between September and October 2025. Participants were selected from a previously monitored cohort according to documented hypoglycemia during HD-day continuous glucose monitoring (CGM). Patients who experienced hypoglycemia on HD received a 15 g oral glucose solution 1.5 h after starting HD. Blood glucose levels were then monitored via CGM. Paired pre-post comparisons were performed at the patient level. Blood glucose and pressure fluctuations were analyzed.

**Results:**

Compared with the pre-intervention period, the incidence of hypoglycemia, time below range, and coefficient of variation were significantly lower during the entire HD day, during the HD session, and within 2 h post-HD (all *P* < 0.05). Mean blood glucose levels were significantly higher after the intervention (*P* < 0.05). The incidence of hypotension and systolic/diastolic blood pressure did not differ significantly at any time point during HD (0–4) or before and after the intervention (all *P*>0.05).

**Conclusions:**

Oral supplementation with 15 g of glucose solution administered 1.5 h into HD was associated with a short-term reduction in hypoglycemia in older patients with diabetes undergoing HD using glucose-free dialysate, without an observed increase in hypotension.

**Clinical trial registration:**

## 1 Introduction

Diabetes is a major cause of chronic kidney disease (CKD) and is now the single leading cause of end-stage renal disease (ESRD), accounting for approximately 44% of patients initiating renal replacement therapy worldwide. Compared with patients without diabetes, those with diabetes undergoing hemodialysis (HD) have a poorer prognosis—including a higher incidence of cardiovascular complications, increased hospitalization, and higher 30-day readmission—and long-term mortality rates owing to large blood glucose fluctuations and heightened susceptibility to hypoglycemia (1).

HD-associated hypoglycemia is more prevalent in patients with diabetes, accounting for 24–56% of cases (2). Most episodes are asymptomatic, and hypoglycemia frequently persists even when glucose-containing dialysates are used (3, 4). Consistent with this, our previous multicenter research demonstrated significant glycemic fluctuations and hypoglycemia prevalence on HD days among older patients with diabetes undergoing HD, particularly during or post-HD (4, 5).

Asymptomatic hypoglycemia in older adults is clinically important because it is closely associated with an increased risk of falls, cardiovascular events, cognitive impairment, and elevated all-cause mortality, as well as higher hospitalization rates. Therefore, effective and rigorous blood glucose monitoring is crucial in this population (6–8).

Conventional monitoring methods, including point blood glucose measurements, self-monitoring of blood glucose, and glycated hemoglobin (HbA1c) measurements, have inherent limitations (9). Continuous glucose monitoring (CGM) provides real-time and continuous data on glycemic fluctuations, thereby assisting healthcare providers in formulating personalized interventions to stabilize blood glucose levels. Our previous study and other relevant research have indicated that CGM may be a valuable tool for personalized treatment and management (3, 4). The Kidney Disease: Improving Global Outcomes guidelines specify that CGM can prevent further hyperglycemia- or hypoglycemia-induced renal damage in patients with diabetes and CKD, especially those with significant glycemic fluctuations and whose blood glucose cannot be effectively controlled by conventional glucose monitoring methods (10).

Strategies to mitigate HD-associated hypoglycemia typically involve the use of a glucose-containing dialysate or food intake during HD. However, both approaches have limitations. Glucose-containing dialysates are prone to bacterial contamination and pose storage challenges (11), and evidence regarding their efficacy in reducing hypoglycemia is inconsistent (9). Although early food intake during HD may mitigate hypoglycemia (12), it can also trigger intradialytic hypotension (IDH), with the risk increasing with calorie intake. Patients who consume >200 kcal during an HD session have twice the incidence of hypotension compared with those who do not eat during HD (13). IDH is a serious complication associated with an increased risk of major adverse events (14).

Currently, no standardized guidelines exist regarding the timing and type of food intake during HD (15); however, the American Association of Clinical Endocrinology Clinical Practice Guidelines recommend the administration of 15–20 g of oral glucose to conscious patients with hypoglycemia (16), providing a potential targeted approach that avoids full meals.

Based on this rationale, we conducted a single-arm, self-controlled, pre-post pilot study of 12 patients with HD-related hypoglycemia. A retrospective continuous glucose monitoring (r-CGM) system was used to compare blood glucose levels and blood pressure fluctuations before and after the intervention, with the aim of providing evidence to support hypoglycemia prevention and management in older patients with diabetes receiving HD. Given that all enrolled participants received glucose-free dialysate, this intervention strategy is of greatest clinical relevance for dialysis centers where glucose-free dialysate is still routinely applied or clinically indicated.

## 2 Methods

### 2.1 Study population

**Figure 1:**
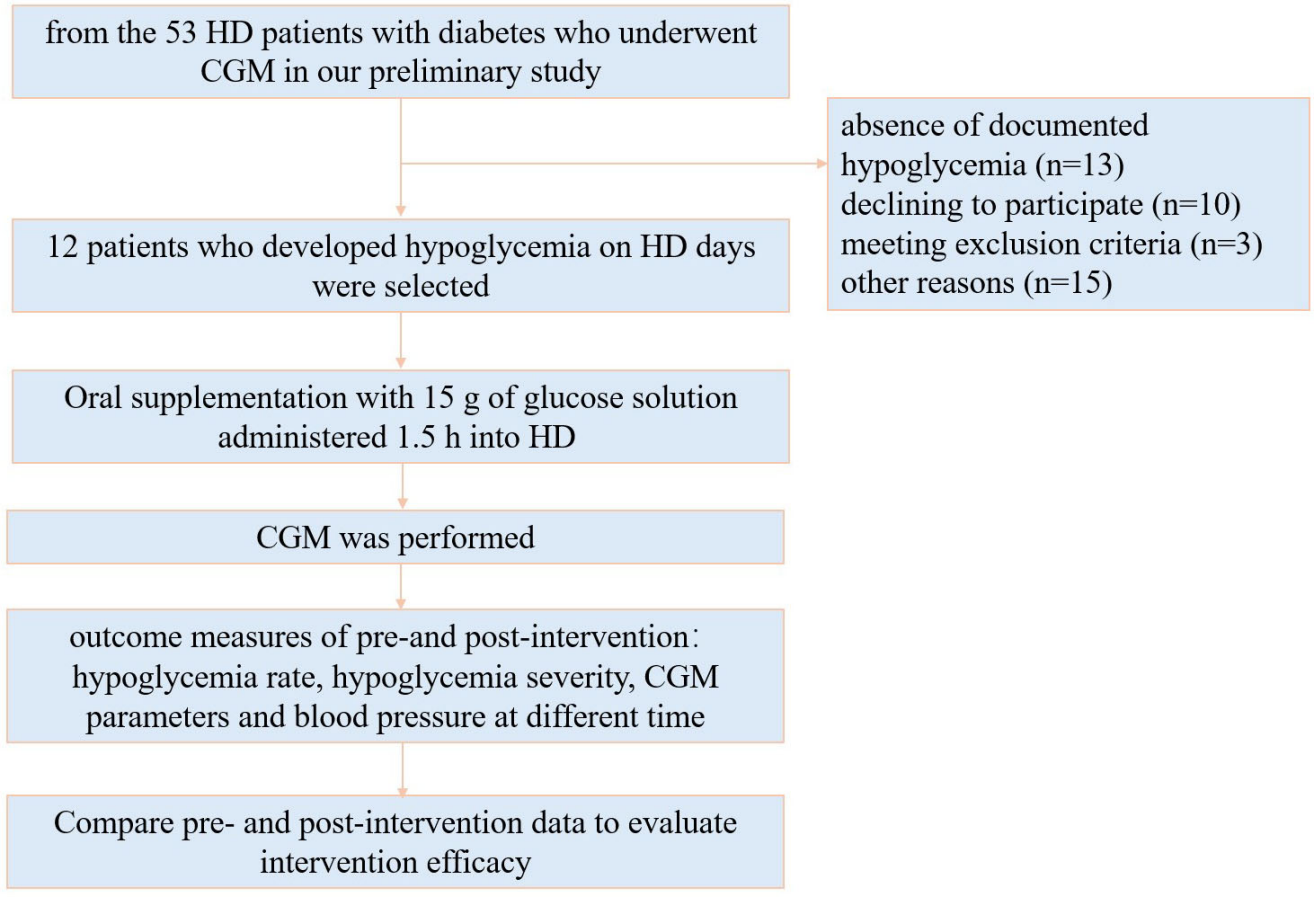
Flow diagram of cohort creation.

Twelve older adults with diabetes who experienced hypoglycemia on HD were enrolled at PLA General Hospital between September and October 2025 (this population was selected from our previous study). Of the 53 CGM-monitored patients in the original cohort, 12 patients who had documented hypoglycemia during HD-day CGM monitoring and who provided written informed consent were enrolled in the intervention study (**Figure 1**) . The pre-intervention data referred to the HD session immediately preceding the intervention session in the same patient, and the intervention was implemented during a single HD session. Accordingly, this study evaluated the acute short-term effect of a single intradialytic glucose administration rather than repeated treatment over time. The diabetes diagnostic criteria followed the 2021 American Diabetes Association (ADA) standards: fasting plasma glucose (FPG) ≥7.0 mmol/L (126 mg/dL) after ≥8 h fasting, 2 h plasma glucose ≥11.1 mmol/L during an oral glucose tolerance test, random plasma glucose ≥11.1 mmol/L, or HbA1c ≥6.5% (17). Hypoglycemia was defined as a blood glucose level <3.9 mmol/L [70 mg/dL] (18). For CGM-based analyses, hypoglycemia was defined as at least one glucose value <3.9 mmol/L during the observation period.

The inclusion criteria were as follows: patients aged ≥60 years diagnosed with diabetes who had experienced hypoglycemia on HD days, had undergone HD treatment for >3 months, with an HD frequency of 3 times/week, and each session lasting 4 h. The exclusion criteria were as follows: use of hormonal drugs or history of blood transfusion within 6 months, acute diabetic complications (diabetic ketoacidosis and diabetic hyperosmolar coma), uncontrolled concurrent inflammatory events, and symptomatic hypoglycemia.

This study was approved by the Ethics Committee of PLA General Hospital (S2023-725-01). All participants volunteered to participate and signed an informed consent form.

### 2.2 Clinical data

The ages of all patients and their general data, including diabetes duration, HD duration, body mass index, and HbA1c level, were collected from medical records. Information on antidiabetic treatment was also collected.

All patients underwent routine HD. The HD sessions were arranged in two daily time windows, morning (08:00–12:00) or afternoon (13:00–17:00). For all participants, glucose-free dialysate was administered throughout the procedure, with a blood flow rate of 240–300 mL/min, dialysate flow rate of 500–800 mL/min, and dialyzer membrane area ranging from 1.4 to 1.8 m². The dialysate temperature was maintained at 36 °C, and the dialysate contained sodium 138 mmol/L, potassium 2 mmol/L, calcium 1.5 mmol/L, magnesium 0.5 mmol/L, chloride 109 mmol/L, acetate 3 mmol/L, and bicarbonate 32 mmol/L.

### 2.3 Research methods

This study used a single-arm, self-controlled, pre-post pilot design. General data were collected from all enrolled patients. At 1.5 h into HD, patients were orally administered 30 mL of a 50% glucose solution, equivalent to 15 g of glucose. The timing of glucose administration (1.5 h into HD) was selected pragmatically based on prior CGM observations from our group indicating that glucose levels often declined progressively during HD and that hypoglycemic events commonly occurred after the early phase of dialysis. Therefore, this time point was intended to provide preventive carbohydrate supplementation before the period of greater hypoglycemia risk. To improve the palatability, a solution with a concentration of approximately 17% was mixed with 50 mL of mineral water in a disposable paper cup. Subsequently, the head of the bed was elevated to 15°–30°, and the patients were instructed to drink the solution within 3 min under direct supervision of the dialysis nurse, who visually confirmed completion. No obvious gastrointestinal adverse reactions were observed in any of the patients. Adverse events were monitored via direct observation during HD and routine nursing inquiries.

Assuming a post-intervention hypoglycemia incidence rate of 35% (3), α=0.01, power 1-β=0.9, the sample size calculated using PASS15 software was N = 9. Assuming a 20% dropout rate, the total required sample size was calculated to be 12 patients. Given the pilot nature of the study, the small sample size, and the paired single-arm design, this study should be interpreted as exploratory rather than confirmatory.

The primary endpoint of the study was the occurrence of hypoglycemia on the HD day after the intervention compared with the pre-intervention HD day. Secondary outcomes included time below range <3.9 mmol/L (TBR^3.9^), mean blood glucose (MBG), coefficient of variation (CV), time in range (TIR), time above range >13.9 mmol/L (TAR^13.9^), and blood pressure-related outcomes during HD. Analyses across multiple time windows were considered exploratory.

### 2.4 Continuous glucose and blood pressure monitoring

r-CGM was performed for 2–6 days before and after the intervention, and for 1–3 days on both HD and non-HD days. In compliance with standardized protocols, glucose sensor probes were implanted by trained registered nurses 2 h before the start of dialysis. The r-CGM system recorded the average value every 5 min for a total of 288 glucose measurements over 24 h. CGM monitoring was performed for 6 days before the intervention and 2–6 days after the intervention for each patient.

A total of 35 HD sessions before and 14 sessions after the intervention were included in the analysis for each patient. After the measurement period, the glucose-monitoring results were imported into the software for analysis. The CGM metrics included MBG, defined as the average glucose level during a specified monitoring period; CV, defined as the standard deviation divided by the mean glucose level and used as an index of glycemic variability; TBR^3.9^, defined as the percentage of time with glucose levels <3.9 mmol/L (70 mg/dL), reflecting the burden of hypoglycemia; TIR, defined as the percentage of time with glucose levels between 3.9 and 10.0 mmol/L (70–180 mg/dL), reflecting the proportion of time spent within the target glucose range; and TAR^13.9^, defined as the percentage of time with glucose levels >13.9 mmol/L (250 mg/dL), reflecting the proportion of time spent above the target glucose range.

For CGM-based analyses, individual sensor readings were not treated as independent observations. Instead, CGM data were summarized into patient-level metrics within each predefined study period and intradialytic time window, and these summarized values were used for analysis.

Blood pressure was measured at HD initiation and at hourly intervals thereafter through hour 4 by the dialysis nurse using an automated sphygmomanometer (Omron, Japan). For analyses of intradialytic hypotension across prespecified HD intervals, the unit of analysis was the patient. For each interval, percentages were calculated as the number of patients who experienced IDH during that interval divided by the total number of patients evaluated.

IDH was defined according to the 2005 National Kidney Foundation Kidney Disease Outcomes Quality Initiative guidelines as a decrease in either SBP of ≥20 mmHg or mean arterial pressure ≥10 mmHg leading to symptoms (19). Clinical symptoms and intervention-related details during hypotensive episodes were not systematically recorded in a standardized manner.

### 2.5 Statistical analysis

Data were entered independently in parallel by two individuals using the EpiData system. Analyses were performed using IBM SPSS Statistics for Windows, version 26.0 (IBM Corp., Armonk, NY, USA). Normally distributed data are expressed as means ± standard deviation (SD), non-normally distributed data as medians (P25, P75), and count data as [n (%)]. Since this was a self-controlled pre-post study, paired analyses were applied throughout. Normally distributed continuous variables were compared using the paired t-test, whereas non-normally distributed continuous variables were compared using the Wilcoxon signed-rank test. Paired categorical variables, including IDH within each prespecified HD interval, were compared using McNemar’s test or exact McNemar’s test, as appropriate. A two-sided *P* value <0.05 was considered statistically significant.

## 3 Results

### 3.1 Baseline patient characteristics

Of the 12 patients included in this study, 8 were male (66.67%) and 4 were female (33.33%). The mean age was 61.67 ± 9.78 years, and the mean duration of diabetes was 18.17 ± 11.10 years. Regarding glucose-lowering treatment, 5 patients were managed with diet alone, 2 received oral hypoglycemic agents, 4 received insulin, and 1 received both oral agents and insulin (**Table 1**).

**Table 1:** General patient data (n=12).

Item	Value
Age (years)	61.67 ± 9.78
Gender (n, %)	
Male	8 (66.67)
Female	4 (33.33)
Body Mass Index (kg/m²)	22.56 ± 4.29
Diabetes Duration (years)	18.17 ± 11.10
Glucose-lowering Regimen (n, %)	
Diet	5 (41.67)
Oral Hypoglycemic Agents	2 (16.67)
Insulin	4 (33.33)
Oral Agents + Insulin	1 (8.33)
HbA1c (%)	6.59 ± 0.81
ALB	40.65(39.65,42.90)

### 3.2 Effects of oral glucose administration on hypoglycemia rates across different periods on the day of HD

Following intervention, hypoglycemia and TBR^3.9^ rates decreased across the entire HD day and all periods. Hypoglycemia rates fell significantly during the entire HD day, during HD, and within 2 h post-HD (*P* = 0.002, *P* < 0.001, and *P=*0.030, respectively). TBR^3.9^ also decreased significantly during the entire HD day [14.59% (2.95, 37.85) vs. 0% (0, 0), *P* = 0.003] and during HD [7.64% (3.13, 11.11) vs. 0% (0, 2.43), *P* < 0.001]. Hypoglycemia severity declined significantly throughout all episodes (*P* = 0.003 and *P* < 0.001, respectively). As patients were recruited according to baseline confirmed hypoglycemia, the decreased incidence of hypoglycemic events post oral glucose could be partially explained by spontaneous variability and regression to the mean instead of intervention efficacy alone. All hypoglycemic episodes remained asymptomatic (**Table 2**).

**Table 2:** Comparison of hypoglycemia outcomes across different periods on the dialysis day before and after the intervention (n=12). .

Item	Period	Pre-intervention	Post-intervention	Test statistic	P value
Hypoglycemia rate, n (%)	HD period (4 h)	11 (91.67)	2 (16.67)	7.11	0.004
	Post-HD period (2 h)	7 (58.33)	1 (8.33)	4.167	0.031
	Night period (6 h)	3 (25.00)	1 (8.33)	0.25	0.625
	Entire day (24 h)	12 (100.00)	4 (33.33)	6.125	0.013
Hypoglycemia severity (mmol/L)	HD period (4h)	3.40 (3.00, 3.70) 95%CI (3.26~3.35)	3.70 (3.50, 3.80) 95%CI (3.59~3.69)	-7.236	<0.001
	Post-HD period (2 h)	3.20 (2.90, 3.70) 95%CI (3.14~3.32)	3.60 (3.50, 3.80) 95%CI (3.49~3.67)	-4.404	<0.001
	Night period (6 h)	3.30 (3.10, 3.70) 95%CI (3.26~3.40)	3.60 (3.30, 3.70) 95%CI (3.48~3.55)	-2.964	0.003
	Entire day (24 h)	3.40 (3.00, 3.70) 95%CI (3.29~3.36)	3.60 (3.53 3.80) 95%CI (3.61~3.68)	-9.655	<0.001
TBR (%)	HD period (4 h)	14.59 (2.95, 37.85) 95%CI (8.73~33.55)	0.00 (0.00, 0.00) 95%CI (-1.15~4.28)	-2.934	0.003
	Post-HD period (2 h)	6.95 (0.00, 19.44) 95%CI (0.88~29.68)	0.00 (0.00, 0.00) 95%CI (-2.70~12.42)	-0.845	0.398
	Night period (6 h)	0.00 (0.00, 0.52) 95%CI (-4.50~17.73)	0.00 (0.00, 0.00) 95%CI (-0.83~2.22)	-1.095	0.273
	Entire day (24 h)	4.75 (2.35, 11.08) 95%CI (2.80~12.58)	0.00 (0.00, 0.02) 95%CI (0.00~0.02)	-3.061	0.002

Note: Data are presented as n (% of 12 patients) or median (P25, P75), as appropriate. Percentages indicate the proportion of patients who experienced at least one hypoglycemic episode during each prespecified period. The HD period refers to the 4h HD session, the post-HD period refers to the first 2 hours after completion of HD, the night period refers to the predefined 6-hour nocturnal interval, and the entire day refers to the full 24-hour monitoring period on HD day. Since this was a self-controlled pre-post study, repeated measurements within the same patient were not treated as independent observations. Paired categorical comparisons were performed using McNemar’s test or exact McNemar’s test, as appropriate, and paired continuous/non-normally distributed data were compared using the Wilcoxon signed-rank test. HD, hemodialysis.TBR,time below range.

### 3.3 Effects of oral glucose administration on blood glucose fluctuations across different periods on the day of HD

**Figure 2:**
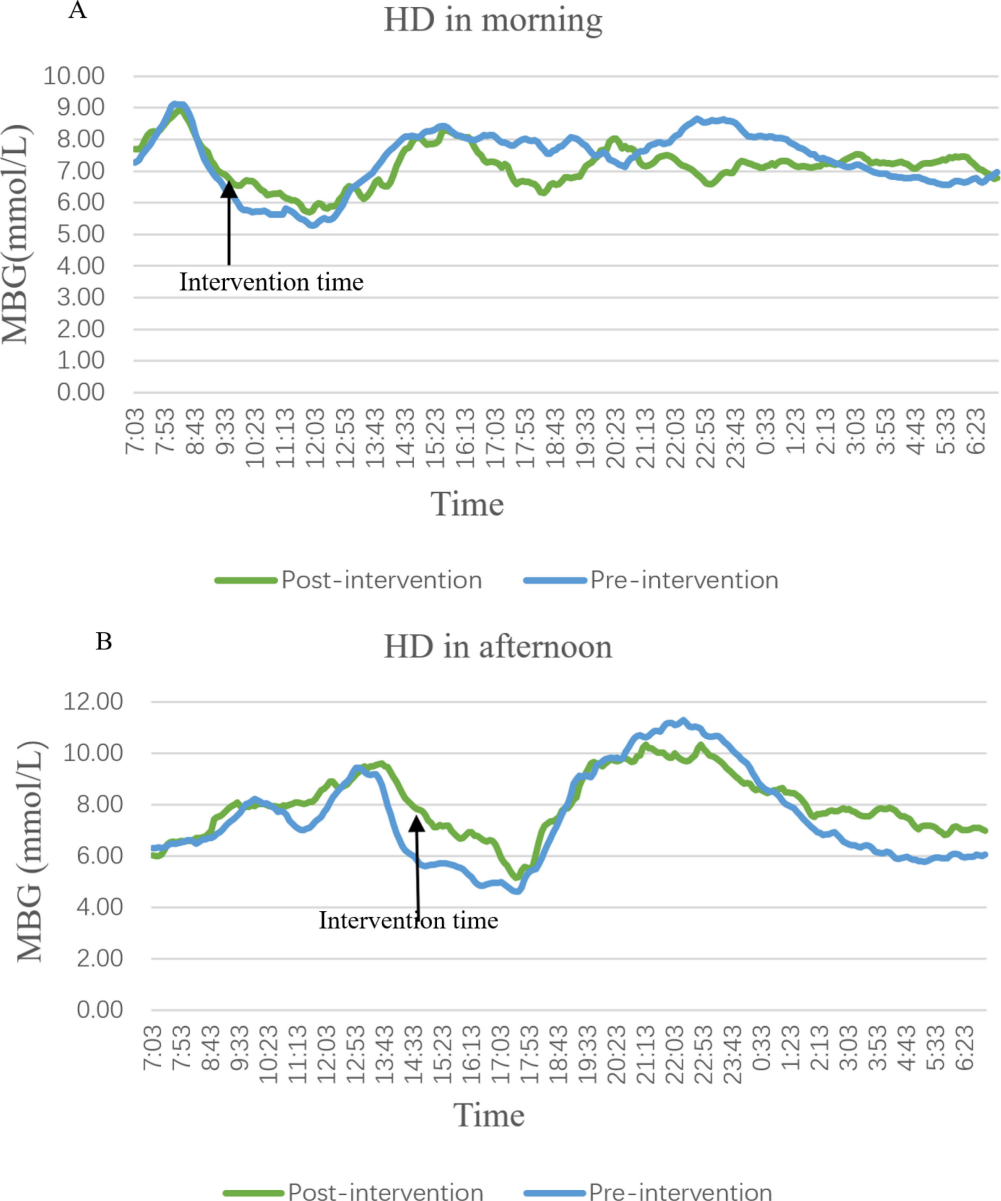
Temporal glucose profiles on the pre-intervention and post-intervention HD days. Note: **(A)** Morning HD. **(B)** Afternoon HD. The blue line represents the pre-intervention data, and the green line represents the post-intervention data. Each MBG value corresponds to the mean blood glucose level for a 1-hour interval.

After the intervention, MBG increased significantly across the entire HD day (7.39 ± 0.95 vs. 8.99 ± 0.74 mmol/L, *P* < 0.001), the HD session (6.06 ± 1.45 vs. 7.30 ± 1.28 mmol/L, *P* = 0.003), and within 2 h post-HD (6.86 ± 2.41 vs. 8.32 ± 2.05 mmol/L, *P* = 0.010). Minimum glucose levels also increased significantly during the HD session, within 2 h post-HD, and over the entire HD day (all *P* < 0.05). Conversely, the CV significantly decreased across the entire HD day (31.96 ± 8.59 vs. 18.57 ± 5.12, *P* < 0.001), during the HD session [22.41% (17.11, 26.92) vs. 17.59% (13.70, 20.43), *P* = 0.012], within 2 h post-HD [21.36% (16.50, 30.54) vs. 11.74% (6.99, 19.03), *P* = 0.016], and during the night [10.95% (8.53, 19.66) vs. 8.96% (6.80, 16.42), *P* = 0.002] (**Table 3**). To further illustrate these temporal glucose fluctuations, CGM-derived temporal glucose profiles were plotted for the pre- and post-intervention HD days (**Figure 2**). In both morning and afternoon HD sessions, glucose levels changed dynamically during and after HD, and the profiles differed between the pre- and post-intervention days. These data complement the MBG values reported at selected post-dialysis time points and provide a more complete visualization of temporal glucose trends. Although the intervention was associated with an increase in mean blood glucose, no significant increase in TAR13.9 was observed in any analyzed period, suggesting that the short-term reduction in hypoglycemia was not accompanied by an apparent increase in marked hyperglycemia in this small pilot sample.

**Table 3:** Comparison of glycemic metrics across different periods before and after the intervention (n=12).

Item	Period	Pre-intervention	Post-intervention	t/z	P value
MBG (mmol/L)	HD period (4 h)	6.06 ± 1.45 95%CI (5.14~6.98)	7.30 ± 1.28 95%CI (6.49~8.11)	-3.764	0.003
	Post-HD period (2 h)	6.86 ± 2.41 95%CI (5.33~8.39)	8.32 ± 2.05 95%CI (7.02~9.62)	-3.112	0.010
	Night period (6 h)	7.03 ± 2.14 95%CI (5.68~8.39)	7.87 ± 2.49 95%CI (6.29~9.45)	-1.658	0.126
	Entire day (24 h)	7.39 ± 0.95 95%CI (6.78~8.00)	8.99 ± 0.74 95%CI (8.52~9.46)	-5.687	<0.001
Max (mmol/L)	HD period (4 h)	9.03 ± 2.32 95%CI (7.55~10.51)	9.53 ± 1.93 95%CI (8.31~10.76)	-1.080	0.303
	Post-HD period (2 h)	8.96 ± 2.92 95%CI (7.10~10.82)	9.37 ± 2.79 95%CI (7.60~11.15)	-0.980	0.348
	Night period (6 h)	9.21 ± 2.90 95%CI (7.37~11.05)	9.77 ± 3.61 95%CI (7.47~12.06)	-0.755	0.466
	Entire day (24 h)	12.93 ± 2.08 95%CI (11.61~14.25)	12.30 ± 2.50 95%CI (10.71,13.89)	1.318	0.214
Min (mmol/L)	HD period (4 h)	3.98 ± 1.27 95%CI (3.17,4.78)	4.97 ± 1.14 95%CI (4.25~5.70)	-2.858	0.016
	Post-HD period (2 h)	4.84 ± 2.03 95%CI (3.55,6.13)	5.48 ± 2.03 95%CI (4.61,6.37)	-1.636	0.130
	Night period (6 h)	5.47 ± 1.74 95%CI (4.37,6.58)	6.30 ± 2.17 95%CI (4.93~7.68)	-1.373	0.197
	Entire day (24 h)	3.48 ± 0.79 95%CI (2.98~3.98)	4.53 ± 0.87 95%CI (3.98~5.08)	-3.318	0.004
TIR(%)	HD period (4 h)	72.92 (50.00, 89.06) 95%CI (58.67~84.39)	90.63 (78.65, 99.65) 95%CI (75.97~97.07)	-2.401	0.016
	Post-HD period (2 h)	75.00 (50.35, 88.20) 95%CI (50.00~83.57)	77.08 (59.38, 100.00) 95%CI (59.03~92.36)	-0.863	0.388
	Night period (6 h)	88.89 (68.23, 100.00) 95%CI (63.39~95.49)	95.83 (65.97, 100.00) 95%CI (55.42~99.91)	-0.296	0.767
	Entire day (24 h)	76.51 ± 14.99 95%CI (66.99~86.03)	81.56 ± 17.23 95%CI (70.61~92.50)	-1.438	0.178
TAR (%)	HD period (4 h)	0.00 (0.00, 1.04) 95%CI (0.42~2.62)	0.00 (0.00, 0.00) 95%CI (-1.46~3.89)	0.000	1.000
	Post-HD period (2 h)	0.00 (0.00, 8.33) 95%CI (-1.34~10.14)	0.00 (0.00, 0.00) 95%CI (-4.17~11.11)	-0.845	0.398
	Night period (6 h)	0.00 (0.00, 0.00) 95%CI (-2.64~7.04)	0.00 (0.00, 0.00) 95%CI (-2.73~8.98)	-1.342	0.180
	Entire day (24 h)	1.45 (0.00, 7.04) 95%CI (0.51~6.80)	0.00 (0.00, 4.25) 95%CI (-0.72~7.61)	-0.338	0.735
CV (%)	HD period (4 h)	22.41 (17.11, 26.92) 95%CI (17.64~32.52)	17.59 (13.70, 20.43) 95%CI (14.72~20.00)	-2.510	0.012
	Post-HD period (2 h)	21.36 (16.50, 30.54) 95%CI (14.41~23.06)	11.74 (6.99, 19.03) 95%CI (11.01~16.28)	2.856	0.016
	Night period (6 h)	10.95 (8.53, 19.66) 95%CI (8.85~19.68)	8.96 (6.80, 16.42) 95%CI (7.12~16.68)	-3.059	0.002
	Entire day (24 h)	31.96 ± 8.59 95%CI (26.50~37.41)	18.57 ± 5.12 95%CI (15.32~21.82)	7.082	<0.001

Data are presented as mean ± SD or median (P25, P75), as appropriate. The HD period refers to the 4-hour hemodialysis session, the post-HD period refers to the first 2 hours after completion of HD, the night period refers to the predefined 6-hour nocturnal interval, and the entire day refers to the full 24-hour monitoring period on the HD day. Since this was a self-controlled pre-post study, paired comparisons were performed using the paired t-test or Wilcoxon signed-rank test, as appropriate. HD, hemodialysis; MBG, mean blood glucose; CV, coefficient of variation; TBR, time below range; TIR, time in range.

### 3.4 Effects of oral glucose administration on blood pressure during HD

The proportion of patients who experienced IDH within each prespecified HD interval did not differ significantly between the pre-intervention and post-intervention sessions (**Table 4**; all P>0.05). Systolic and diastolic blood pressure values at each prespecified HD time point were not significantly different before and after the intervention (**Supplementary Table 2**; all P>0.05).

**Table 4:** Comparison of the proportion of patients with IDH across prespecified HD intervals before and after the intervention (n=12).

HD interval	pre-intervention	post-intervention	McNemar χ²	P value
(0-1h)	5 (41.67%)	6 (50.00%)	0.333	1.000
(1-2h)	5 (41.67%)	3 (25%)	0.125	0.727
(2-3h)	1 (8.33%)	3 (25%)	0.25	0.625
(3-4h)	2 (16.67%)	2 (16.67%)	0.00	1.000

Data are presented as number (% of 12 patients). Percentages indicate the proportion of patients who experienced intradialytic hypotension within each prespecified HD interval. Each interval was analyzed separately in the pre- and post-intervention periods. Since this was a self-controlled pre-post study, paired categorical comparisons were performed using McNemar’s test or exact McNemar’s test, as appropriate. HD, hemodialysis.

## 4 Discussion

This single-arm, self-controlled, pre-post pilot study evaluated the effect of administering 15 g of oral glucose solution to patients 1.5 h into HD. This intervention significantly decreased hypoglycemia and TBR^3.9^ rates throughout the HD day and at all specific time points while reducing hypoglycemia severity. Additionally, MBG increased after the intervention during the entire HD day, the HD session, and within 2 h post-HD, whereas CV decreased across the same periods. As patients were recruited according to baseline confirmed hypoglycemia, the decreased incidence of hypoglycemic events post oral glucose could be partially explained by spontaneous variability and regression to the mean instead of intervention efficacy alone. Although the intervention improved hypoglycemia-related measures, the observed increase in mean blood glucose suggests a potential trade-off. While this increase may be clinically acceptable in the context of hypoglycemia prevention, its possible impact on hyperglycemia exposure and time above range should also be taken into consideration. These findings suggest that a small dose of oral glucose supplement administered during HD may be an effective and practical strategy for reducing intradialytic hypoglycemia and improving short-term glycemic stability.

The kidney plays a central role in glucose regulation and mediates glucose reabsorption, utilization, and gluconeogenesis. Among patients with advanced CKD or ESKD, those with diabetes exhibit significant alterations in glucose and insulin metabolism (20). Hypoglycemia is often overlooked in this population despite its association with major adverse outcomes. Reduced estimated glomerular filtration rates (eGFRs) result in an altered metabolism and changes in the safety profile of hypoglycemic agents, and lower eGFR is linked to higher risks of hypoglycemia, cardiovascular events, and mortality (21, 22). The incidence of hypoglycemic crisis in adults aged ≥18 years with diabetes and ESKD is approximately threefold higher than that of hyperglycemic crisis (23).

Multiple interacting factors contribute to hypoglycemia, including reduced food intake and impaired renal gluconeogenesis owing to the loss of functional proximal tubular cells. Notably, patients with diabetes and concurrent kidney disease have diminished renal drug clearance, and even standard doses of insulin or other hypoglycemic agents may result in higher blood concentrations. Finally, HD can increase the risk of hypoglycemia owing to the low glucose concentration in the dialysate, intradialytic glucose transfer, increased erythrocyte glucose uptake, and prolonged half-life of the hypoglycemic drugs (24). Clinically, prompt detection and correction of hypoglycemia, particularly asymptomatic episodes, are crucial for preventing severe adverse events.

Patients with ESRD undergoing HD have an elevated risk of hypoglycemia via multiple pathways, including reduced renal gluconeogenesis, impaired insulin and hypoglycemic agent metabolism and clearance, protein-energy wasting, and intradialytic glucose transfer to red blood cells (25). Given the well-established adverse effects of hypoglycemia on cardiovascular outcomes, survival, and quality of life, frequent, convenient, and accurate blood glucose measurements are urgently required.

HbA1c is the gold standard for long-term glycemic control in the general population, but it shows discrepancies in patients with CKD (including HD) owing to decreased hematopoietic function, shortened red blood cell lifespan, and reduced glycation rates in these patients. Anemia and erythropoietin use may result in falsely low HbA1c levels, whereas elevated blood urea nitrogen and metabolic acidosis may lead to falsely high values, limiting HbA1c use in kidney disease [especially advanced CKD or HD] (24). Although glycated albumin (GA) is unaffected by red blood cell lifespan, anemia, or erythropoietin use, its measurement is affected by chronic inflammation, nephrotic-range proteinuria, and hypoalbuminemia (25). Therefore, GA monitoring is not recommended in patients with CKD.

In this context, CGM offers important advantages. CGM captures real-time glucose fluctuations and can detect clinically silent hypo- and hyperglycemia (26). International guidelines recommend CGM for glycemic control assessments in patients undergoing HD, especially in those with hypoglycemia unawareness or a high risk of recurrent hypoglycemia (27). CGM provides summary data on TIR, TAR, and TBR as glycemic variability indicators, which guide treatment adjustments by evaluating overall glycemic control and identifying glucose trends and hypoglycemia risk. In a UK-based multicenter study of 10,370 patients across 102 hospitals, flash CGM use improved glycemic control, enhanced hypoglycemia awareness, reduced diabetes-related distress, and decreased hospital admissions compared with the baseline (28).

In the present study, an oral glucose solution was administered to patients during HD to improve hypoglycemia without increasing the incidence of IDH, thereby providing a potential strategy for IDH prevention. IDH is a common and severe complication in patients undergoing HD, affecting approximately 20–30% of patients (29). IDH is associated with adverse outcomes, including myocardial infarction, heart failure hospitalization, volume overload, cardiovascular mortality, and all-cause mortality (30, 31). Additionally, IDH may induce dizziness, fatigue, headache, nausea, and vomiting during HD, impairing a patient’s quality of life and potentially leading to premature HD termination, thus reducing HD adequacy.

Current IDH prevention strategies include lowering the dialysate temperature, adjusting the ultrafiltration rates, using a sodium gradient, and implementing high-flux hemofiltration (31). However, the evidence for these interventions remains limited. Another approach for ameliorating IDH is dietary intake during HD. Patients undergoing HD generally also exhibit malnutrition and hypoalbuminemia; while protein meal supplementation during HD may confer some benefits, it can also trigger IDH, presumably owing to splanchnic blood flow redistribution. Thus, controversy persists regarding the overall effects of intradialytic food intake on IDH: some studies suggest benefits, whereas others show an increased risk of IDH owing to splanchnic blood flow redistribution (29, 32). However, large-scale, long-term studies on the benefits of food intake during HD are lacking.

Thus, an unmet clinical need exists regarding interventions to ensure adequate energy supply during HD, reduce the incidence of hypoglycemia, and avoid hypotension. In response to this need, the present study selected an oral glucose solution owing to its unique advantages: unlike sodium chloride solution, it has favorable palatability, and its osmotic effect dissipates rapidly following glucose clearance and metabolism. Our findings align with prior research showing that intravenous glucose infusion during HD prevents symptomatic IDH without causing severe hypertensive episodes (33).

Compared with meal-based nutritional interventions, oral glucose solution confers multiple practical merits in HD settings. First, it delivers a fixed, low-dose carbohydrate supplement, thereby minimizing interindividual variations attributed to meal components, appetite status, and eating rate. Second, oral glucose solution features rapid intestinal absorption and convenient administration under routine nursing supervision. Third, in contrast to solid meals, it imposes a lower burden on splanchnic blood perfusion and may reduce the risk of intradialytic hemodynamic instability; nevertheless, this presumption warrants further validation in large-scale clinical trials.

Notably, glucose-free dialysate is not routinely used in all countries or centers, and glucose-containing dialysate is generally preferred where feasible to reduce the risk of intradialytic hypoglycemia. Therefore, the findings of the present study may be more applicable to settings in which glucose-free dialysate is still used or remains necessary and may not be directly generalizable to centers routinely using glucose-containing dialysate.

Some limitations should be acknowledged. First, the study population included a highly selected cohort: only 12 of 53 patients from the original CGM-monitored cohort were included because hypoglycemia was documented during a limited observation period. Although appropriate for a proof-of-concept pilot study, this enrichment strategy introduces substantial selection bias, may exaggerate the apparent benefit of the intervention, and limits generalizability. In addition, the absence of hypoglycemia during a short CGM monitoring period should not be interpreted as indicating low future risk, given the marked day-to-day variability in intradialytic glycemia related to dialysis timing, food intake, glucose-lowering therapy, ultrafiltration volume, and other intercurrent factors. Second, the observed post-intervention improvement may partly reflect regression to the mean, as participants were enrolled based on confirmed hypoglycemia before oral glucose administration. The reduction in hypoglycemic events should therefore be interpreted as preliminary and hypothesis-generating rather than definitive evidence of efficacy. Third, several potentially important determinants of intradialytic hypoglycemia and hypotension—including dietary intake, glucose-lowering therapy, ultrafiltration-related parameters, dialysis prescription, and antihypertensive medication use—were not fully standardized or incorporated into the analysis, leaving the possibility of residual confounding. Moreover, because data on symptoms and interventions during hypotensive episodes were limited, these events were identified primarily by blood pressure criteria, which may have constrained assessment of their clinical severity. Fourth, despite meeting the age-based inclusion criterion, the cohort should not be assumed to represent a clearly geriatric or frail population, as frailty, functional status, cognitive status, and geriatric syndromes were not formally evaluated. Fifth, multiple outcomes were analyzed across predefined time windows; these exploratory analyses may have increased the risk of type I errors. Sixth, nutritional status and pre-dialysis food intake were not systematically assessed, although both may have influenced intradialytic hypoglycemia and hypotension. Accordingly, the potential contribution of nutritional factors could not be fully evaluated. In addition, several dialysis-related variables that may influence metabolic and hemodynamic responses, such as ultrafiltration volume/rate, dry weight, interdialytic weight gain, and changes in antihypertensive treatment, were not collected, which should be addressed in future studies.

In conclusion, oral administration of 15 g glucose during HD was associated with a short-term reduction in hypoglycemia in older patients with diabetes undergoing HD with glucose-free dialysate. No increase in hypotension was observed; however, the study was not powered to establish safety. These findings should therefore be considered preliminary and hypothesis-generating. Larger multicenter studies are required to confirm these findings.

## Data Availability

The raw data supporting the conclusions of this article will be made available by the authors, without undue reservation.
