# Supplementary material for: Short-term effect of oral glucose solution in older patients with hypoglycemia undergoing hemodialysis
Source: Front Endocrinol (Lausanne). 2026 Jun 19;17:1861404. doi: 10.3389/fendo.2026.1861404 (PMC13341799; doi:10.3389/fendo.2026.1861404)
Supplement: Supplementary file 1 [file Table1.docx]

| **Table S1 Blood glucose indicators of individuals** | | | | | | |
| --- | --- | --- | --- | --- | --- | --- |
| **Subject** | **Group** | **MBG (mmol/L)** | **z** | **P** | **CV (%)** | **TBR (%)** |
| 1 | Pre | 6.30(5.10,7.80)  95%CI (6.09~6.54) | -4.750 | ＜0.001 | 28.32% | 5.90% |
|  | Post | 6.80(5.80,7.78)  95%CI (6.68~7.05) |  |  | 17.57% | 0.00% |
| 2 | Pre | 5.90(5.40,6.70)  95%CI (5.93~6.10) | -13.380 | ＜0.001 | 19.91% | 1.97% |
|  | Post | 7.70(6.80,8.80)  95%CI (7.69~7.98) |  |  | 9.24% | 0.00% |
| 3 | Pre | 8.90(5.30,11.30)  95%CI (7.85~8.57) | -6.679 | ＜0.001 | 42.14% | 14.70% |
|  | Post | 8.95(6.43,11.98)  95%CI (9.17~10.09) |  |  | 20.56% | 1.04% |
| 4 | Pre | 4.80(4.20,6.68) 95%CI (5.21~5.62) | -13.732 | ＜0.001 | 27.56% | 3.47% |
|  | Post | 7.10(6.10,9.40) 95%CI (7.15~7.72) |  |  | 14.27% | 4.51% |
| 5 | Pre | 6.70(5.60,8.08) 95%CI (6.79~7.42) | -10.542 | ＜0.001 | 38.16% | 3.59% |
|  | Post | 7.90(6.00,9.30) 95%CI (8.00~8.60) |  |  | 28.65% | 0.00% |
| 6 | Pre | 6.90(5.40,9.50) 95%CI (7.60~8.62) | -10.456 | ＜0.001 | 43.19% | 9.95% |
|  | Post | 10.20(8.20,12.18) 95%CI (9.88~10.61) |  |  | 21.07% | 0.00% |
| 7 | Pre | 6.30(5.10,7.30) 95%CI (7.08~7.43) | -1.855 | 0.064 | 23.89% | 7.99% |
|  | Post | 7.00(5.90,7.90) 95%CI (6.85~7.19) |  |  | 18.20% | 2.43% |
| 8 | Pre | 5.80(5.40,6.70) 95%CI (5.97~6.23) | -7.099 | ＜0.001 | 26.51% | 11.47% |
|  | Post | 6.80(5.30,7.70) 95%CI (6.79~7.20) |  |  | 11.87% | 2.43% |
| 9 | Pre | 8.70(6.40,11.00) 95%CI (6.74~7.16) | -14.569 | ＜0.001 | 30.05% | 1.62% |
|  | Post | 9.50(8.50,11.10) 95%CI (9.64~10.07) |  |  | 19.09% | 0.00% |
| 10 | Pre | 5.20(3.90,7.70) 95%CI (5.95~6.89) | -3.329 | 0.001 | 47.38% | 27.78% |
|  | Post | 6.30(4.80,7.80) 95%CI (6.27~6.68) |  |  | 21.29% | 0.00% |
| 11 | Pre | 7.70(6.50,9.50) 95%CI (8.21~8.66) | -0.335 | 0.737 | 28.8% | 3.59% |
|  | Post | 8.75(6.70,9.70) 95%CI (8.18~8.62) |  |  | 22.74% | 0.00% |
| 12 | Pre | 5.90(5.20,7.50) 95%CI (6.35~6.60) | -3.880 | ＜0.001 | 27.56% | 0.23% |
|  | Post | 6.30(5.40,7.50) 95%CI (6.57~6.81) |  |  | 18.30% | 0.00% |

**Table S2 Comparison of** **Blood Pressure at Different Time Points During HD Before and After Intervention (n=12)**

| **Item** | **pre-intervention** | **post-intervention** | **t/z** | **P value** |
| --- | --- | --- | --- | --- |
| SBP (mmHg) |  |  |  |  |
| HD (0h) | 159.00 (143.00, 164.00)  95%CI (145.86~158.21) | 155.00 (142.00, 165.00)  95%CI (149.32~160.81) | -0.676 | 0.499 |
| HD (1h) | 149.00 (132.00, 156.00)  95%CI (136.89~151.36) | 141.00 (126.00, 152.00)  95%CI (132,82~146.41) | -1.607 | 0.108 |
| HD (2h) | 145.00 (136.00, 153.00)  95%CI (138.52~149.67) | 135.00 (127.00, 146.00)  95%CI (132.25~145.05) | -1.307 | 0.191 |
| HD (3h) | 144.42 ± 17.69  95%CI (137.93~150.91) | 136.06 ± 22.48  95%CI (127.82~144.31) | 1.668 | 0.106 |
| HD (4h) | 155.00 (142.00, 161.00)  95%CI (142.95~155.11) | 145.00 (131.00, 156.00)  95%CI (137.57~148.88) | -1.677 | 0.094 |
| DBP (mmHg) |  |  |  |  |
| HD (0h) | 75.84 ± 9.66  95%CI (72.30~79.38) | 76.13 ± 15.58  95%CI (70.41~81.84) | -1.000 | 0.921 |
| HD (1h) | 75.19 ± 9.08  95%CI (71.86~78.53) | 72.55 ± 13.80  95%CI (67.49~77.61) | 0.926 | 0.362 |
| HD (2h) | 77.94 ± 9.42  95%CI (74.48~81.39) | 73.03 ± 12.19  95%CI (69.56~78.50) | 1.429 | 0.163 |
| HD (3h) | 78.00 ± 8.00  95%CI (75.07~80.93) | 73.90 ± 12.66  95%CI (69.29~78.58) | 1.574 | 0.126 |
| HD (4h) | 79.61 ± 8.30  95%CI (76.57~82.66) | 78.84 ± 11.86  95%CI (74.49~83.19) | 0.290 | 0.774 |
| **Note:** Data are presented as mean ± SD or median (P25, P75), as appropriate. Blood pressure was assessed at prespecified time points during HD (0, 1, 2, 3, and 4 h), where 0 h indicates HD initiation. Pre- and post-intervention blood pressure values at each time point were compared using paired t-tests or Wilcoxon signed-rank tests, as appropriate. SBP, systolic blood pressure; DBP, diastolic blood pressure; HD, hemodialysis. | | | | |
